# Feasibility, outcomes, and cost analysis of robotic head and neck surgery in the Public Health System

**DOI:** 10.31744/einstein_journal/2026AO1666

**Published:** 2026-03-27

**Authors:** Marco Aurélio Vamondes Kulcsar, Christiana Maria Ribeiro Salles Vanni, Leandro Luongo de Matos, Lenine Garcia Brandão, Luiz Paulo Kowalski, Claudio Roberto Cernea, Dhara Giovanna Santin de Souza

**Affiliations:** 1 Universidade de São Paulo Instituto do Câncer do Estado de São Paulo Faculdade de Medicina São Paulo SP Brazil Department of Head and Neck Surgery, Instituto do Câncer do Estado de São Paulo, Faculdade de Medicina, Universidade de São Paulo, São Paulo, SP, Brazil.

**Keywords:** Robotics, Robotic surgical procedures, Head and neck neoplasms, Costs and cost analysis, Minimally invasive surgical procedures

## Abstract

**Objective:**

To assess the feasibility and benefits of transoral robotic surgery in the public healthcare system and to conduct a cost analysis of its implementation.

**Methods:**

This prospective, nonrandomized, observational cohort study was conducted over 24 months and included patients with T1-T2 oropharyngeal and supraglottic laryngeal tumors.

**Results:**

Thirty patients were included. The mean surgery duration was 204 min, and the mean hospital stay was 2.1 days. Postoperative complications included one case of bleeding and one case of aspiration. The estimated 5-year survival rate was 77%. Cost analysis revealed a higher initial cost for robotic surgery compared with conventional approaches; however, reduced postoperative complications mitigated the overall expenses.

**Conclusion:**

Transoral robotic surgery is a feasible and safe option within the public healthcare system. It enables highly complex procedures with faster postoperative recovery, lower morbidity, and survival outcomes comparable to conventional approaches.

## Highlights

Transoral robotic surgery applied in patients with T1–T2 oropharyngeal and laryngeal tumors.

Mean hospital stay: 2.1 days.

Minimal postoperative complications.

5-year survival: 77%, with comparable cost-effectiveness.

## In Brief

Transoral robotic surgery is feasible within the public healthcare system, enabling minimally invasive treatment of T1–T2 oropharyngeal and supraglottic laryngeal tumors. It has demonstrated faster recovery, reduced morbidity, and an estimated 5-year survival rate of 77%, with costs comparable to those of conventional surgeries.

## Graphical abstract

**Figure:**
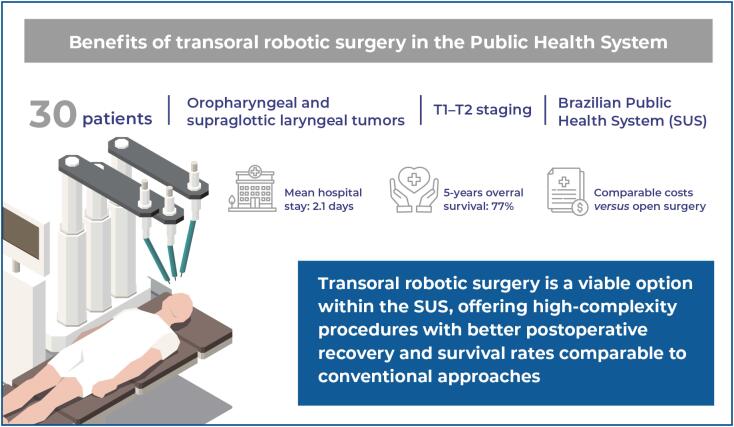

## INTRODUCTION

Owing to technological advancements in recent decades, surgery has undergone significant evolution, particularly in imaging and the development of surgical equipment designed for less invasive techniques such as videolaparoscopy. In head and neck surgery, the advent of microcameras, microscopes, lasers, and refined surgical instruments has enabled the treatment of pharyngolaryngeal pathologies—both benign and malignant—through transoral access, thereby eliminating the need for cervical incisions. These procedures are collectively referred to as transoral surgeries.^(1,2)^

Among the transoral approaches, transoral robotic surgery (TORS) employs robotic systems such as the da Vinci system, manufactured by Intuitive Surgical^®^, and the Flex Robotic system from Medrobotics^®^.^(3)^ The da Vinci system was initially developed for other surgical specialties—urology, gynecology, general surgery, and cardiac surgery—but was later adapted for the treatment of head and neck cancers.^(4)^

In head and neck surgery, the University of Pennsylvania was a key institution responsible for coordinating and developing multicenter research projects on TORS. One of its surgeons pioneered the application of this technique through experimental studies assessing its safety, conducting animal model investigations, introducing it into clinical practice, and reporting experiences with specific procedures. Additionally, he standardized the surgical technique, patient positioning, required materials, instruments, and retractors, as well as the setup of the operating room. As a consultant for Intuitive Surgical®, he also contributed to the publication of the company's official manual, which provides detailed guidelines and requirements for performing TORS.^(5-8)^

Since 2005, numerous studies have highlighted the advantages of TORS over traditional approaches. The use of robotic systems allows surgeons to perform procedures that would otherwise require laparoscopic or transoral endoscopic methods, with the added benefits of stable three-dimensional (3D) visualization and precise instrument control. These systems eliminate physiological tremors and enhance maneuverability across all three spatial dimensions. Additionally, the surgeon operates from a seated console, which improves ergonomic conditions.^(1-2)^

**Figure 1:**
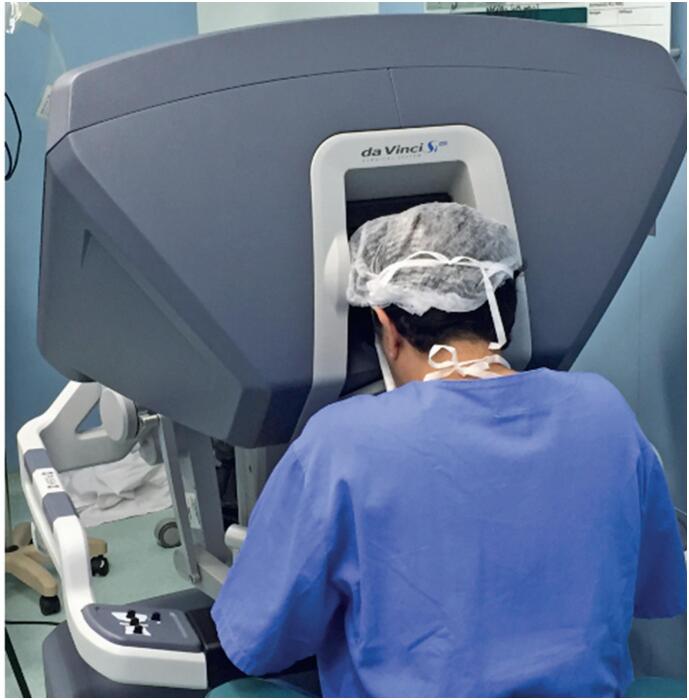
Surgeon's console of the intuitive robotic system

**Figure 2:**
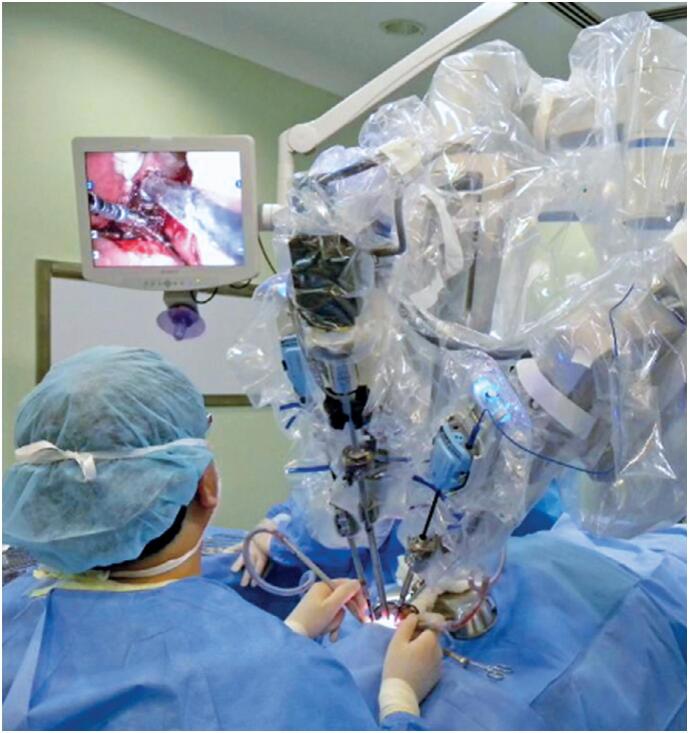
Robotic cart with articulated arms and the assistant's monitor

The robotic system consists of the following elements: a surgeon's console with an integrated 3D stereoscopic viewer (Figure 1), a patient-side cart housing the robotic arms (Figure 2), a video and image capture system, and a monitor for the assistant. The surgeon sits at the console and manipulates the instruments using two master controls, assisted by an operative field assistant. The surgeon's head rests between infrared sensors in the viewer area, allowing 3D visualization of the surgical field through a 3D camera attached to the robotic arm in a central position. The lateral arms hold the surgical instruments and align them within the visual field. Using console controls, the surgeon can manipulate the instrument tips with 360º precision through articulated joints, thereby reproducing the dexterity of conventional surgical techniques.^(1-4,9)^

The electronic components of the robot reduced the hand movements of the surgeon, translating them into proportionally smaller motions at the instrument tips. The video system allows the operating room team to follow the surgical procedures in real time. Robotic instruments offer seven degrees of freedom with multiple adjustable settings, enabling the surgeon to optimize the operative field while eliminating physiological tremors through an electronic filtering system that ensures stability and precision of movement.^(1-4,9)^

Despite these advantages, the system has some drawbacks, including its high cost and the absence of tactile feedback, which prevents the surgeon from differentiating between flexible and rigid tissues by touch. However, high-quality magnified visualization (×10) compensates for this lack of haptic perception. The overall cost includes several factors: purchase of the robotic system, maintenance, specialized instruments, and their limited reusability. The learning curve involves training in system operation, simulated exercises, completion of a written certification examination, cadaver training, animal model practice, and a supervised clinical training period.

Initially, TORS was limited to procedures involving the skull base, pharynx, and larynx. However, as techniques were refined, its application expanded to include thyroidectomy (via the anterior chest wall) and surgeries employing retroauricular and extended cervical approaches. In the pharyngeal and skull base regions, TORS provides access to the nasopharynx, sphenoid sinus, pharyngeal wall, palatine and lingual tonsils, soft palate, tongue base, and piriform sinuses. In the larynx, the da Vinci system enables supraglottic procedures involving the epiglottis and aryepiglottic folds, whereas the Medrobotics system facilitates glottic interventions similar to conventional laryngeal microsurgery.^(10-16)^

**Figure 3:**
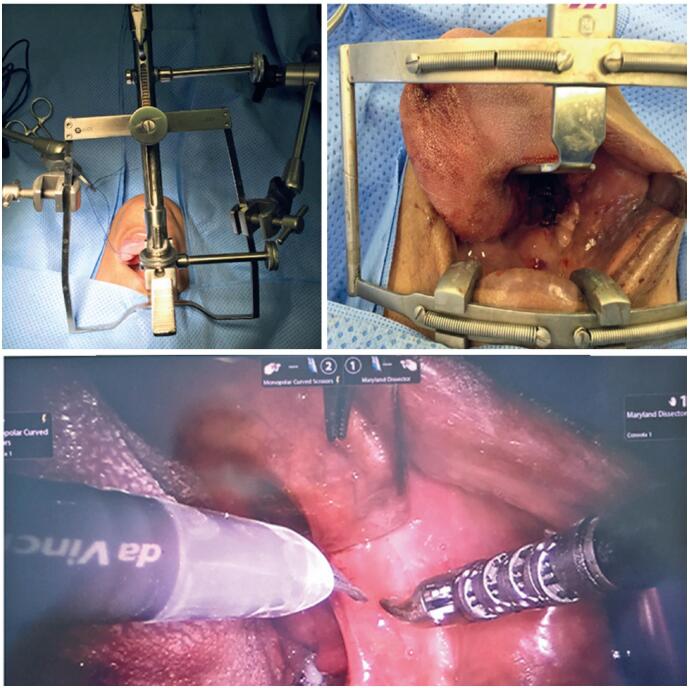
(Top left) FK retractor used for transoral surgery. (Top right) Dingman retractor for transoral surgery with a view of the tonsillar tumor on the right. (Bottom) Robotic instruments within the pharynx

The robotic system can also be applied to benign diseases in these regions, such as pharyngolaryngeal cysts, lingual thyroid, tonsillectomy, and resection of tumors in the parapharyngeal space, such as deep-lobe parotid neoplasms. For malignant lesions, its use is primarily indicated for early-stage T1 and T2 tumors (Figure 3), offering several advantages over conventional treatments that require cervical access, mandibulotomy, or a mid-labial-buccal flap. These conventional approaches are associated with postoperative complications such as scarring, malocclusion, dysphagia, and in many cases, the need for protective tracheostomy.^(15-21)^

Thus, the advantages of robotic surgery include shorter hospital stays, reduced operative time, lower intraoperative blood loss, avoidance of pharyngocutaneous fistula, and faster overall patient recovery.^(22)^

## OBJECTIVE

Given these considerations, this study aimed to demonstrate the applicability of robotic surgery via transoral access within the public health system and to evaluate the benefits associated with its use.

## METHODS

This was a prospective, nonrandomized, observational cohort study involving a series of cases over a 24-month period. The study commenced after ethical approval was granted by the Research Ethics Committee of the *Faculdade de Medicina* of *Universidade de São Paulo* (CAAE: 77422123.8.0000.0068; #6.815.829) and informed consent was obtained from all participants.

The inclusion criteria were as follows: 1) tumors of the oropharynx and supraglottic larynx classified as T1-T2; 2) metastatic tumors of unknown primary origin with positron emission tomography/computed tomography (PET/CT) demonstrating positive uptake in the oropharynx; 3) recurrent malignant tumors of the oropharynx and larynx (epiglottis) after conservative chemoradiotherapy; and 4) neopharyngeal tumors after total laryngectomy.

Exclusion criteria included patients under 18 years, those who declined participation, those lost to follow-up, and those with limited oral opening that prevented positioning of the robotic arms.

The study initially enrolled 34 patients between 2015 and 2017, of whom four were excluded: one due to tumor progression, two because of insufficient exposure of the supraglottic region during surgery, and one owing to arrhythmia during anesthesia induction.

All patients were admitted after evaluation by the head and neck surgery team and staged according to institutional protocols, including clinical assessment, nasofibroscopy, upper digestive endoscopy, and computed tomography.

Surgical procedures were performed using the da Vinci XI robotic system (Intuitive Surgical^®^) with either Dingman or Factory retractors, depending on the case. The robot was coupled with a 0^o^ degree endoscope and two arms using 5-mm cautery forceps—either spatula tip or hook type—assisted by 5-mm Maryland forceps. The procedures followed in internationally established protocols, such as radical palatine tonsillectomy for oropharyngeal tumors.^(5,9)^

Robotic transoral surgery was compared with conventional cervical surgery for tumors of equivalent stage. The comparison included length of hospital stay, intraoperative expenses, and all postoperative outcomes.

## RESULTS

Of the 30 patients evaluated, 22 (73.3%) were male and 8 (26.7%) were female. The mean age was 63.5 years, range 46-85 years.

All patients had good performance status, 29 were classified as Eastern Cooperative Oncology Group (ECOG) 0, and only one as ECOG 1. The mean body mass index was 24.7 (range, 18-33.3). Additionally, 17 patients (56.7%) had a history of tobacco use, whereas the remaining 13 (43.3%) had never smoked.

**Figure 4:**
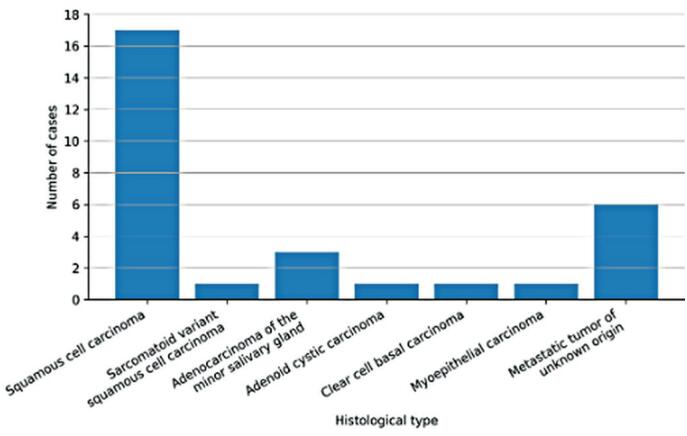
Histological distribution of operated tumor types

The most common histological type was squamous cell carcinoma, observed in 17 patients (56.7%). Surgery was also performed in 1 case (3.3%) of sarcomatoid variant squamous cell carcinoma, 3 cases (10%) of adenocarcinoma of the minor salivary gland, 1 case (3.3%) of adenoid cystic carcinoma, 1 case (3.3%) of clear cell basal carcinoma, and 1 case (3.3%) of myoepithelial carcinoma. In addition, 6 patients (20%) presented with metastatic tumor of unknown origin showing positive 18-fluorine-18 fluorodeoxyglucose PET/CT uptake in the oropharynx (Figure 4).

The most common surgeries performed were oropharyngectomy (26 cases) and epiglottectomy (4 cases). When analyzed by anatomical site, tumor locations were as follows: 12 (40%) in the palatine tonsil, 5 (16.7%) in the base of the tongue, 5 (16.7%) in the soft palate, 6 (20%) in the epiglottis, and 2 (6.6%) in the neopharynx after total laryngectomy. In this series, only 4 cases (13.3%) were HPV-positive by P16 immunohistochemistry, all located in the pharynx.

According to the TNM classification, primary tumor (T) staging was distributed as follows: 15 patients (50%) were T1, 9 (40%) were T2, and 6 (20%) were T0. Regarding nodal status (N), 21 patients (70%) were N0, 1 (3.3%) was N1, 2 (6.7%) were N2a, 3 (10%) were N2b, 1 (3.3%) was N2c, and 2 (6.7%) were N3.

In 10 cases with nodal metastases, neck dissection was performed 10 days before the robotic procedure. In five cases where elective dissection was considered, it was omitted, and sentinel lymph node evaluation was performed, all in tonsillar tumors.

The mean TORS duration was 204 min, and the mean hospital stay was 2.1 days. Preventive tracheostomy was required in eight cases, while a nasoenteral tube was used in 25 cases to facilitate feeding due to the surgical extent and to reduce postoperative pain.

Regarding complications, there was one case of postoperative bleeding requiring reoperation and one case of aspiration, which prolonged hospitalization by an additional 2 days for antibiotic therapy.

The estimated 60-month overall survival was 77%, with four deaths occurring at an average of 12 months due to distant metastasis.

In the cost analysis, robotic buccopharyngectomy (TORS) was associated with a higher mean cost (R$20,094.65) compared with conventional midlabial access with mandibulectomy (R$18,463.00). However, despite this apparent difference of approximately R$1,200.00 in favor of the conventional approach, the large cost variation (standard deviation ≈ 400%) — mainly driven by more frequent postoperative complications — suggests that, in the long term, the robotic approach may greater cost-effectiveness.

## DISCUSSION

The present study found that patients undergoing TORS had shorter hospital stays and lower rates of postoperative complications than those undergoing conventional surgery (mean hospital stay, 4.5 days). These findings align with those of previous studies that demonstrated reduced recovery time, decreased need for tracheostomy, and better preservation of anatomical function.^(23,24)^

The preservation of pharyngolaryngeal function is a clinically relevant outcome. Patients treated with TORS reported less impairment of swallowing and a reduced need for prolonged nutritional support, which favors faster recovery and an earlier return to daily activities.^(22,25)^

Cost variability for TORS is mitigated in part by reductions in complications and length of hospital stay. Additional costs associated with open surgery include prolonged hospitalization, increased antibiotic use, and the need for reoperation for complications; these factors can significantly increase overall hospital expenditures.^(19,26)^

Previous studies have highlighted that the financial benefits of TORS extend beyond individual patients to the healthcare system by reducing rehabilitation and hospital readmission costs.^(2,24)^

Moreover, patient-reported quality of life after TORS is often superior is often superior to that after conventional surgery, and our findings corroborate the existing literature, indicating that TORS offers clinically and economically significant advantages over conventional techniques.^(1,19)^

Despite these advantages, TORS has certain limitations that may impede widespread adoption. The high initial cost of equipment and the need for specialized surgeon training are significant barriers to large-scale implementation, especially in public healthcare systems.^(27,28)^ The learning curve may also be an early obstacle, as procedural accuracy and efficacy improve with the surgeon's experience.^(25)^

Another critical limitation is the absence of tactile (haptic) feedback, which may compromise the surgeon's ability to perceive soft tissues and delicate structures. Advances in robotic technology, including enhanced visualization and improved instrument control, have sought to address this deficiency.^(3,8)^

The continued evolution of robotic systems may increase the adoption of TORS by reducing equipment costs and improving training techniques. Future research should prioritize direct comparisons between TORS and other minimally invasive modalities, focusing on long-term patient quality of life and overall healthcare system cost analyses.^(7,11)^

## CONCLUSION

Transoral robotic surgery is an innovative approach for treating head and neck tumors, particularly early-stage oropharyngeal and supraglottic laryngeal neoplasms. In this study, transoral robotic surgery proved to be a viable and safe treatment option, associated with shorter hospital stays, fewer complications, and improved postoperative quality of life. Despite financial constraints and the procedural learning curve, implementation of transoral robotic surgery is expected to expand as technology advances and surgeon training improves. In addition, we found transoral robotic surgery to be feasible within the public health system because it enables highly complex procedures with lower morbidity, promotes better postoperative outcomes and comparable survival to conventional approaches, and may offer acceptable long-term cost-effectiveness despite higher initial costs. Further research with larger sample cohorts and a randomized design is necessary to validate these findings and to explore additional clinical applications of transoral robotic surgery.

## Data Availability

Data are available to reviewers upon request.
